# On the Use of Side‐Chain NMR Relaxation Data to Derive Structural and Dynamical Information on Proteins: A Case Study Using Hen Lysozyme

**DOI:** 10.1002/cbic.202000674

**Published:** 2020-12-14

**Authors:** Lorna J. Smith, Wilfred F. van Gunsteren, Niels Hansen

**Affiliations:** ^1^ Department of Chemistry University of Oxford Inorganic Chemistry Laboratory South Parks Road Oxford OX1 3QR UK; ^2^ Laboratory of Physical Chemistry Swiss Federal Institute of Technology ETH 8093 Zurich Switzerland; ^3^ Institute of Thermodynamics and Thermal Process Engineering University of Stuttgart 70569 Stuttgart Germany

**Keywords:** averaging time, conformation sampling, nuclear magnetic resonance, S2 order parameters, structure refinement

## Abstract

Values of S2CH and S2NH order parameters derived from NMR relaxation measurements on proteins cannot be used straightforwardly to determine protein structure because they cannot be related to a single protein structure, but are defined in terms of an average over a conformational ensemble. Molecular dynamics simulation can generate a conformational ensemble and thus can be used to restrain S2CH and S2NH order parameters towards experimentally derived target values S2CH (exp) and S2NH (exp). Application of S2CH and S2NH order‐parameter restraining MD simulation to bond vectors in 63 side chains of the protein hen egg white lysozyme using 51 S2CH (exp) target values and 28 S2NH (exp) target values shows that a conformational ensemble compatible with the experimentally derived data can be obtained by using this technique. It is observed that S2CH order‐parameter restraining of C−H bonds in methyl groups is less reliable than S2NH order‐parameter restraining because of the possibly less valid assumptions and approximations used to derive experimental S2CH (exp) values from NMR relaxation measurements and the necessity to adopt the assumption of uniform rotational motion of methyl C−H bonds around their symmetry axis and of the independence of these motions from each other. The restrained simulations demonstrate that side chains on the protein surface are highly dynamic. Any hydrogen bonds they form and that appear in any of four different crystal structures, are fluctuating with short lifetimes in solution.

## Graphical abstract

**Different techniques, same answer**: The application of *S* ^2^ order‐parameter restraining molecular dynamics simulations to the protein HEWL shows that this technique is able to produce a conformational ensemble compatible with experimentally derived side‐chain order parameters and demonstrate that side chains on the protein surface are highly dynamic.

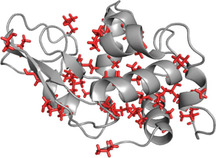

## 1 Introduction

During the past 50 years, the determination of structure of proteins in crystal based on the reflections of X‐rays has become a standard procedure to obtain information on proteins at the atomic level of resolution. Over the past 30 years NMR measurement of proteins in solution has also become a standard technique, not only to obtain information on protein structure, but also on dynamics at the atomic level of resolution. Several quantities that are observable by NMR, such as nuclear Overhauser enhancements (NOEs), ^3^ *J* couplings or chemical shifts, only provide local structural information. Only residual dipolar couplings (RDCs) do provide longer‐range information in terms of the average (relative) directions of bond vectors throughout a molecule. In contrast, X‐ray diffraction of crystals yields non‐local information and, in addition, its information density, that is, the ratio of the number of independent measured values of observable quantities for a molecule and the number of independent molecular degrees of freedom, is much higher than that of NMR experiments on proteins in solution. On the other hand, NMR measurements may provide dynamic information in the form of relaxation data, for example, expressed as *S* ^2^ order parameters, and of all techniques available to obtain information on proteins in solution, NMR shows the highest information density.^[1]^

All techniques to derive structural information from the measurement of observable quantities *Q* make use of a relation of *Q* to structure *r*, a function *Q*(*r*).^[1]^ Since virtually all experimental techniques measure an average ⟨*Q*⟩_space,time_ of *Q* over the molecules (space) in the test tube and over a time window determined by the type of experiment, the derivation of structural information from a set of ⟨*Q*⟩ values should account for the averaging involved in the measurement. This can be done by applying multi‐molecule averaging^[2]^ or by time‐averaging^[3]^ structure refinement instead of the commonly used single‐structure refinement technique. Application of time‐averaging structure refinement to proteins based on X‐ray data,[^4^, ^5^] NMR NOE[^6^, ^7^] or ^3^ *J* coupling^[8]^ data showed the protein structural variation to be much larger than that observed using single‐structure refinement techniques.^[1]^

For observable quantities *Q*, such as X‐ray reflection intensities *I_hkl_*, NOEs (when represented as atom‐atom distance bounds), ^3^ *J* couplings or chemical shifts, it is possible to formulate a function *Q*(*r*) relating a *Q* value to a particular structure *r*. RDCs may be directly related to structure by assuming a single alignment tensor representing the anisotropic rotational distribution of the molecule, which is, unfortunately, unknown. For other observable quantities, such as *S* ^2^ order parameters, the function relating *Q* to *r* involves some average over the Boltzmann ensemble of structures in solution, *Q*(⟨*f*(*r*)⟩), where *f* denotes the function of *r* that is being averaged.^[1]^ This means that structure refinement based on such quantities must involve the averaging ⟨*f*(*r*)⟩ in addition to the averaging ⟨*Q*(⟨*f*(*r*)⟩)⟩.^[9]^

*S* ^2^ order parameters are commonly derived from an analysis of NMR relaxation data using a so‐called “model free” model^[10]^ and can be calculated as an ensemble‐ or time‐average of a function of the three Cartesian coordinate components of the ^13^C−^1^H or ^15^N−^1^H bond vector.^[11]^ They are commonly interpreted as a measure of the geometrical restriction (*S* ^2^=0: no restriction; *S* ^2^=1: complete restriction) of the directions of the mentioned bond vectors on a time scale faster than the stochastic rotational tumbling of the molecule in solution, for proteins of the order of nanoseconds. For relatively stable proteins, order parameters involving backbone atoms lie generally in the range 0.75<*S* ^2^<0.95. Exceptions occur in longer loops connecting secondary structure elements (*S* ^2^ values as low as 0.5) and for thermodynamic conditions, such as low pH, that are known to often destabilise proteins. For bond vectors in side chains, values as low as 0.05 can be found.

MD simulations have reproduced experimentally derived order‐parameter values for bond vectors involving backbone atoms with some success.[^12^, ^13^] For side chains, this is more challenging,[^14^, ^15^, ^16^, ^17^] because of the flexibility of side chains and of the multiple hydrogens bound to the ^13^C atom in a CH_3_ ‐group or bound to the ^15^N atom in a NH_2_− group. This suggests the use of *S* ^2^ order‐parameter structure refinement using time averaging in order to obtain a conformational ensemble compatible with the order‐parameter data. The technique of time‐averaging order‐parameter structure refinement has been tested on the backbone ^15^N−^1^H order parameters of the B3 domain of protein G,^[9]^ and subsequently applied to backbone ^15^N−^1^H order parameters of the protein IL‐4 at pH 6 to detect inconsistencies and model flaws regarding complementary sets of NMR data,^[18]^ and applied to backbone ^15^N−^1^H order parameters of the protein hGH at pH 2.7 in order to explain the occurrence of low order‐parameter values in the middle of stable helices.^[19]^

The application of *S* ^2^ order‐parameter restraining to CH_3_ and NH_2_ moieties in protein side chains is more challenging than to backbone NH groups. The multiple hydrogens cause ambiguity regarding peak assignments, which requires additional assumptions. The directional variability of bond vectors in side chains is generally larger than for backbone N−H or C_α_−H vectors, leading to a greater variety of and smaller order‐parameter values. Third, averaging over side‐chain motions may take longer to converge than over limited backbone motion in a stable protein.

Here the application of *S* ^2^ order‐parameter restraining to side‐chain NH, NH_2_ and CH_3_ moieties of the protein hen egg white lysozyme (HEWL)[^20^, ^21^] is investigated. An earlier comparison of the experimentally derived *S* ^2^ values with those obtained from short, 1 ns unrestrained MD simulations showed a poor relation between simulation and experiment,^[14]^ which could be due to the short simulation time period or force‐field deficiencies, assuming no flaws in the experimental data. Use of an improved force field, of much longer sampling, and of *S* ^2^ order‐parameter restraining might generate a conformational ensemble compatible with these and other experimental data on HEWL.

HEWL is one of the proteins most studied. Several X‐ray crystal structures are available, and sets of NOE data,^[22]^ of ^3^ *J* couplings,^[23]^ RDC values,^[24]^ and of *S* ^2^ order parameters,[^20^, ^21^] measured at a variety of thermodynamic conditions. Here the configurational ensembles, generated in unrestrained and in order‐parameter restrained MD simulations, will be used to calculate side‐chain ^13^C−^1^H and ^15^N−^1^H order‐parameter values, which are compared to values obtained from NMR measurements at pH 3.5.[^20^, ^21^]

The side‐chain *S* ^2^ values were separated into two groups, one of C−H values and another of N−H values. By separately restraining these subsets of *S* ^2^ values, it could be investigated whether restraining one subset would improve the agreement with values derived from experiment for the other subset of *S* ^2^ values. Four X‐ray crystal structures were used in the simulations and for comparison, in order to delineate the influence of a particular starting structure on the generated configurational ensemble.

The simulated configurational ensembles were also used to calculate NOE atom‐atom distances that were compared to a set of NOE atom‐atom distance bounds derived from experiment at pH 3.8.^[22]^ A set of side‐chain ^3^ *J*_HαHβ_ couplings derived from experiment is available.[^22^, ^23^] The 26 ^3^ *J*_HαHβ_ couplings in side chains for which *S* ^2^ order‐parameter values derived from experiment are available, were used for comparison. They regard *χ*_1_ torsional angles close to the backbone. A comparison of simulated with experimentally derived ^3^ *J*_HαHβ_ coupling values is not straightforward though, because the Karplus relation ^3^ *J*(*θ*) that connects a torsional angle *θ* to a ^3^ *J* coupling, possesses a rather large uncertainty of 1–2 Hz,^[25]^ small ^3^ *J* coupling values (≈4 Hz) are difficult to determine precisely from spectra, and ^3^ *J* couplings in the range 5–8 Hz may result from averaging over long time periods (microseconds). The set of RDCs for side chains of HEWL^[24]^ was not used for comparison, because they strongly depend on the solvent environment in the measurement.^[26]^

## Computational Methods

Energy minimisations and molecular dynamics simulations were performed using the GROMOS bio‐molecular simulation software.[^27^, ^28^, ^29^]

### Molecular model

The protein was modelled using the GROMOS bio‐molecular force field 54 A7.[^30^, ^31^] In view of the pH used in the experimental NMR measurements, pH 3.5, only Glu35 was protonated and His was doubly protonated.^[32]^ The simple point charge (SPC) model^[33]^ was used to describe the solvent molecules in the rectangular periodic box. To compensate for the overall positive charge of the protein, 10 Cl^−^ ions were included in the solution. All bond lengths and the bond angle of the water molecules were kept rigid with a relative geometric precision of 10^−4^ using the SHAKE algorithm,^[34]^ allowing for a 2 fs MD time step in the leap‐frog algorithm^[35]^ used to integrate the equations of motion. For the non‐bonded interactions a triple‐range method^[36]^ with cut‐off radii of 0.8/1.4 nm was used. Short‐range van der Waals and electrostatic interactions were evaluated every time step based on a charge‐group pair list.^[29]^ Medium‐range van der Waals and electrostatic interactions, between pairs at a distance larger than 0.8 nm and shorter than 1.4 nm, were evaluated every fifth time step (10 fs), at which time point the pair list was updated, and kept constant between updates. Outside the larger cut‐off radius a reaction‐field approximation[^37^, ^38^] with a relative dielectric permittivity of 61^[39]^ was used. Minimum‐image periodic boundary conditions were applied.

### Simulation set‐up

Four X‐ray crystal structures were used as initial structures for the energy minimisations followed by MD simulations.

1. Structure 2VB1 from the Protein Data Bank (PDB),^[40]^ derived from a triclinic unit cell at 0.065 nm resolution at *T*=100 K. It contains multiple side‐chain conformations for 46 residues.
2. Structure 4LZT from the PDB, derived from a triclinic unit cell at 0.095 nm resolution at *T*=295 K. It contains multiple side‐chain conformations for 8 residues.
3. Structure 1IEE from the PDB, derived from a tetragonal unit cell at 0.094 nm resolution at *T*=110 K. It contains multiple side‐chain conformations for 33 residues.
4. Structure 1AKI from the PDB, derived from a orthorhombic unit cell at 0.15 nm resolution at *T*=298 K. It contains no multiple side‐chain conformations.

For the initial structures the side‐chain conformation with the highest occupancy was chosen.

An initial structure was first energy minimised in vacuo to release possible strain induced by small differences in bond lengths, bond angles, improper dihedral angles, and short non‐bonded contacts between the force‐field parameters and the X‐ray structure. Subsequently, the protein was put into a rectangular box filled with water molecules, such that the minimum solute‐wall distance was 1.0 nm, and water molecules closer than 0.23 nm from the solute were removed. This resulted in boxes with 12157 water molecules for the initial protein structures. In order to relax unfavourable contacts between atoms of the solute and the solvent, a second energy minimisation was performed for the protein in the periodic box with water while keeping the atoms of the solute harmonically position‐restrained^[29]^ with a force constant of 25 000 kJ mol^−1^ nm^−2^.

The resulting protein‐water configuration (i. e., coordinates) was used as initial configuration for the MD simulation. In order to avoid artificial deformations in the protein structure due to relatively high‐energy atomic interactions still present in the system, the MD simulation was started at *T*=60 K and then the temperature was slowly raised to *T*=308 K. Initial atomic velocities were sampled from a Maxwell distribution at *T*=60 K. The equilibration scheme consisted of five short 20 ps simulations at temperatures 60, 120, 180, 240 and 308 K at constant volume. During the first four of the equilibration periods, the solute atoms were harmonically restrained to their positions in the initial structures with force constants of 25 000, 2500, 250, and 25 kJ mol^−1^ nm^−2^. The temperature was kept constant using the weak coupling algorithm^[41]^ with a relaxation or coupling time *τ*_Τ_=0.1 ps. Solute and solvent were separately coupled to the heat bath. Following this equilibration procedure, the simulations were performed at a reference temperature of 308 K and a reference pressure of 1 atm. The pressure was kept constant using the weak coupling algorithm^[41]^ with a coupling time *τ*_p_=0.5 ps and an isothermal compressibility *κ*_T_=4.575×10^−4^ (kJ mol^−1^ nm^−3^)^−1^. The centre of mass motion of the system was removed every 1000 time steps (2 ps).

### Order‐parameter restraining

Two sets of ^13^C−^1^H and ^15^N−^1^H side‐chain order‐parameter target values S2XY (exp)[^20^, ^21^] for restraining^[9]^ were used, see Tables 1, 2–3.

**Table 1:** S2CH values (51) derived from relaxation measurements and from four unrestrained MD simulations starting from four X‐ray crystal structures, and the mean of the latter four values and the root‐mean‐square deviation (RMSD) from it. Order‐parameter target values larger than 0.95 were set to 0.95 (second column between brackets). Values differing more than 0.2 from the experimental value (0.95 in case the experimental value is 1) are denoted using italics.

Residue and methyl group	Experimental value^[21]^	MD simulation					
		*2VB1*	*4LZT*	*1IEE*	*1AKI*	Mean	RMSD
Val2 CG2	0.598	*0.39*	0.51	0.50	0.43	0.46	0.05
Leu8 CD1	0.767	0.58	0.60	0.61	*0.53*	0.58	0.03
Leu8 CD2	0.803	0.63	*0.60*	0.64	*0.57*	0.61	0.03
Ala9 CB	1.0 (0.95)	0.93	0.93	0.93	0.93	0.93	0.003
Ala10 CB	0.901	0.91	0.92	0.92	0.92	0.92	0.003
Ala11 CB	0.861	0.91	0.93	0.92	0.92	0.92	0.01
Met12 CE	0.812	*0.33*	*0.47*	*0.26*	*0.58*	*0.41*	0.12
Leu17 CD1	0.630	0.46	0.61	*0.33*	0.52	0.48	0.10
Leu17 CD2	0.632	0.49	0.58	*0.37*	0.55	0.50	0.08
Leu25 CD1	1.0 (0.95)	*0.40*	*0.57*	*0.30*	*0.41*	*0.42*	0.10
Leu25 CD2	0.609	0.42	0.56	*0.32*	*0.37*	0.42	0.09
Val29 CG1	0.871	*0.57*	*0.66*	*0.61*	*0.58*	*0.61*	0.03
Val29 CG2	0.791	*0.57*	0.65	0.60	*0.57*	0.60	0.03
Ala31 CB	0.98 (0.95)	0.94	0.94	0.93	0.93	0.94	0.004
Thr43 CG2	0.361	*0.68*	*0.78*	*0.79*	*0.62*	*0.72*	0.07
Thr47 CG2	0.327	*0.73*	*0.71*	*0.67*	*0.68*	*0.70*	0.02
Thr51 CG2	0.778	*0.49*	0.58	0.64	*0.46*	*0.54*	0.07
Ile55 CG2	0.739	*0.49*	0.70	0.61	0.71	0.63	0.09
Ile55 CD	0.323	*0.55*	*0.57*	*0.58*	*0.72*	*0.61*	0.07
Leu56 CD1	0.734	0.79	0.76	0.80	0.77	0.78	0.02
Leu56 CD2	0.681	0.75	0.72	0.76	0.71	0.74	0.02
Ile58 CG2	1.0 (0.95)	0.84	0.86	0.86	0.88	0.86	0.01
Ile58 CD	0.160	*0.81*	*0.76*	*0.75*	*0.82*	*0.78*	0.03
Thr69 CG2	0.98 (0.95)	*0.72*	0.77	*0.71*	*0.74*	*0.73*	0.02
Leu75 CD1	0.590	0.62	0.73	0.62	*0.37*	0.58	0.13
Ile78 CG2	0.810	0.85	0.72	0.70	*0.52*	0.70	0.12
Ile78 CD	0.416	0.43	0.36	0.42	0.35	0.39	0.03
Leu83 CD1	0.884	*0.68*	*0.65*	0.77	*0.53*	*0.66*	0.09
Leu83 CD2	0.783	0.66	0.61	0.74	*0.52*	0.63	0.08
Leu84 CD1	1.0 (0.95)	*0.46*	*0.66*	*0.66*	*0.63*	*0.60*	0.08
Leu84 CD2	0.879	*0.45*	*0.63*	*0.64*	*0.60*	*0.58*	0.08
Ile88 CG2	0.697	0.55	0.81	0.62	0.80	0.70	0.11
Ile88 CD	0.722	*0.27*	*0.45*	*0.34*	*0.37*	*0.36*	0.06
Thr89 CG2	1.0 (0.95)	*0.71*	*0.64*	*0.66*	*0.72*	*0.68*	0.03
Ala90 CB	0.919	0.91	0.92	0.91	0.92	0.92	0.004
Val92 CG1	0.764	0.63	0.83	0.75	0.85	0.76	0.09
Val92 CG2	0.707	0.61	0.81	0.74	0.83	0.75	0.09
Ala95 CB	0.680	*0.94*	*0.93*	*0.94*	*0.94*	*0.94*	0.01
Ile98 CG2	0.740	0.90	0.83	0.87	0.85	0.86	0.02
Ile98 CD	0.815	0.89	0.85	0.82	0.85	0.85	0.03
Val99 CG1	0.487	*0.85*	*0.78*	0.68	0.52	*0.71*	0.12
Val99 CG2	0.517	*0.85*	*0.80*	0.68	0.53	0.71	0.12
Met105 CE	0.630	0.80	*0.36*	0.56	*0.39*	0.52	0.18
Ala107 CB	0.832	0.88	0.88	0.87	0.80	0.86	0.03
Val109 CG2	0.354	0.36	0.38	0.38	0.51	0.41	0.06
Val120 CG1	0.660	0.69	0.55	0.61	0.55	0.60	0.06
Ala122 CB	0.879	0.78	0.85	0.75	0.82	0.80	0.03
Ile124 CG2	0.753	0.75	0.56	0.67	0.72	0.68	0.07
Ile124 CD	0.351	0.48	0.39	0.55	*0.56*	0.50	0.07
Leu129 CD1	0.525	*0.12*	*0.15*	*0.16*	*0.20*	*0.16*	0.03
Leu129 CD2	0.507	*0.11*	*0.12*	*0.11*	*0.19*	*0.13*	0.03

**Table 2:** S2NH values (11) for Trp (NE1‐HE1) and Arg (NE‐HE) side chains derived from relaxation measurements and from four unrestrained MD simulations starting from four X‐ray crystal structures, and the mean of the latter four values and the root‐mean‐square deviation (RMSD) from it. Values differing more than 0.2 from the experimental value are denoted using italics.

Residue	Experimental	MD simulation					
	value^[20]^	*2VB1*	*4LZT*	*1IEE*	*1AKI*	Mean	RMSD
Trp28	0.90	0.88	0.84	0.85	0.87	0.86	0.02
Trp62	0.41	*0.73*	*0.66*	*0.75*	0.57	*0.68*	0.07
Trp63	0.88	0.83	0.81	0.85	0.78	0.82	0.03
Trp108	0.87	0.87	0.80	0.89	*0.61*	0.79	0.11
Trp111	0.88	0.83	0.78	0.78	0.80	0.80	0.02
Trp123	0.85	0.70	0.68	*0.61*	0.66	0.66	0.03
Arg61	0.28	0.22	0.30	0.33	0.32	0.29	0.04
Arg73	0.12	0.24	0.17	*0.40*	0.19	0.25	0.09
Arg112	0.31	0.28	0.16	0.23	0.18	0.21	0.05
Arg114	0.27	0.13	0.32	0.19	0.19	0.21	0.07
Arg125	0.05	0.12	0.14	0.10	0.14	0.13	0.02

**Table 3:** S2NH values (17) for Asn (ND2‐HD21, ‐HD22) and Gln (NE2‐HE21, ‐HE22) side chains derived from relaxation measurements and from four unrestrained MD simulations starting from four X‐ray crystal structures, and the mean of the latter four values and the root‐mean‐square deviation (RMSD) from it. The experimental values correspond to either HD/E21 or HD/E22.^[20]^ The assignment in the second column is based on the best agreement with the values of the *MD*_*2VB1* simulation (third column). Values differing more than 0.2 from the experimental value are denoted using italics.

Residue	Experimental value^[20]^	MD simulation					
		2VB1	4LZT	1IEE	1AKI	Mean	RMSD
Asn19 HD21	0.43	0.49	0.46	0.34	0.42	0.43	0.06
Asn19 HD22		0.24	0.31	0.23	0.23	0.25	0.03
Asn27 HD21		0.86	0.78	0.79	0.82	0.81	0.03
Asn27 HD22	0.72	0.82	0.60	0.62	0.70	0.69	0.09
Asn37 HD21	0.51	0.37	0.36	0.41	0.43	0.39	0.06
Asn37 HD22		0.21	0.21	0.16	0.25	0.21	0.03
Asn39 HD21	0.74	0.80	0.79	0.74	0.79	0.78	0.02
Asn39 HD22		0.61	0.59	0.54	0.59	0.58	0.03
Gln41 HE21		0.31	0.42	0.26	0.39	0.35	0.06
Gln41 HE22	0.19	0.21	0.21	0.16	0.24	0.21	0.03
Asn44 HD21		0.75	0.75	0.58	0.68	0.69	0.07
Asn44 HD22	0.51	0.71	0.68	0.60	0.62	0.65	0.04
Asn46 HD21		0.85	0.84	0.86	0.80	0.84	0.02
Asn46 HD22	0.62	0.82	0.68	0.58	0.74	0.71	0.09
Gln57 HE21	0.82	0.79	0.74	0.72	0.67	0.73	0.04
Gln57 HE22		0.76	0.54	0.64	0.37	0.58	0.14
Asn59 HD21		0.92	0.92	0.90	0.91	0.91	0.01
Asn59 HD22	0.78	0.90	0.89	0.86	0.87	0.88	0.02
Asn65 HD21		0.76	0.64	0.66	0.73	0.70	0.05
Asn65 HD22	0.57	0.42	*0.28*	*0.33*	*0.25*	*0.32*	0.06
Asn74 HD21	0.74	0.66	*0.52*	0.60	0.54	0.58	0.05
Asn74 HD22		0.41	0.31	0.36	0.37	0.36	0.04
Asn77 HD21		0.54	0.48	0.47	0.34	0.46	0.07
Asn77 HD22	0.24	0.31	0.22	0.28	0.22	0.26	0.04
Asn93 HD21	0.59	0.53	0.63	0.52	0.72	0.60	0.08
Asn93 HD22		0.34	0.32	0.30	0.40	0.34	0.04
Asn103 HD21		0.72	0.33	0.49	0.36	0.48	0.15
Asn103 HD22	0.26	*0.61*	0.18	0.33	0.20	0.33	0.17
Asn106 HD21	0.58	0.68	0.44	0.67	0.47	0.57	0.11
Asn106 HD22		0.46	0.24	0.47	0.29	0.37	0.10
Asn113 HD21	0.47	0.40	0.65	*0.79*	0.58	0.61	0.14
Asn113 HD22		0.21	0.31	0.55	0.45	0.38	0.13
Gln121 HE21	0.36	0.34	0.31	0.50	0.39	0.39	0.07
Gln121 HE22		0.18	0.09	0.36	0.21	0.21	0.10

1. A set of 51 S2CH (exp) values for CH_3_ moieties in 30 residues,^[21]^
2. A set of 28 S2NH (exp) values for NH and NH_2_ moieties in six Trp, five Arg, fourteen Asn and three Gln residues.^[20]^

**Figure 1:**
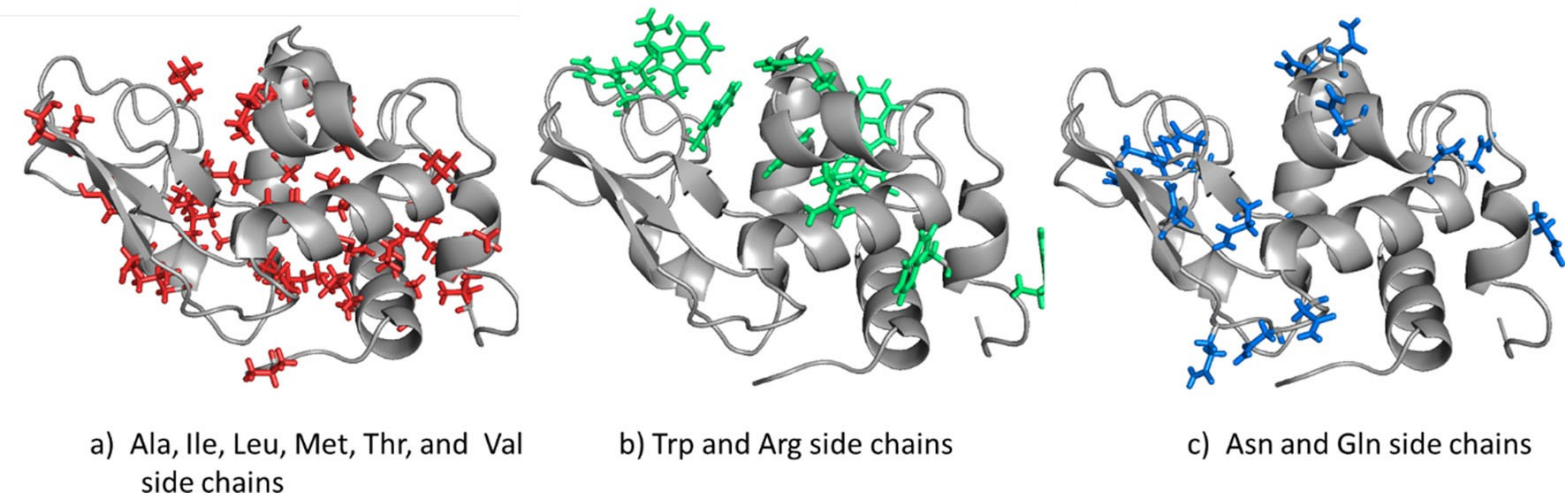
Ribbon pictures of the structure of HEWL with explicit side chains for which *S* ^2^(exp) order‐parameter values derived from relaxation measurements are available. Left: Ala, Ile, Leu, Met, Thr and Val side chains; middle: Arg and Trp side chains; right: Asn and Gln side chains.

The distribution of these *S* ^2^ values over the protein is shown in Figure 1.

For the Asn and Gln residues, one S2NH (exp) value per NH_2_ group was available. This required the assignment to one of the two NH1 and NH2 bond vectors. This was done by calculating S2NH1 (MD) and S2NH2 (MD) values from the unrestrained simulation *MD*_*2VB1* starting from the 2VB1 X‐ray structure and then selecting the N−H vector with its S2NH (MD) value closest to S2NH (exp) for restraining. A corresponding procedure was used to assign experimentally unassigned S2CG1 and S2CG2 values for Val residues and S2CD1 and S2CD2 values for Leu residues.

For an ideal methyl group with equal and fixed C−H bond lengths and H−C−H bond angles in which rotation around the symmetry axis occurs uniformly, the order parameter for the C−H bond vector is given by^[15]^

(1) S2rot=((3cos2β-1)/2)2

where β is the angle between a C−H vector and the symmetry axis, which can be considered equal to the C−C bond vector of the bond to the C‐atom adjacent to the CH_3_ group. When in addition the rotational motion around the C−C axis is independent of the motion of the C‐axis itself, one may factorise their contributions,

(2) S2CH=S2CCS2rot

When β=109.5°, one has^[15]^ S2rot =0.111. Thus the methyl group restraining is applied to the C−C bond vector and the target value is

(3) S2CC(exp)=S2CH(exp)/0.111

For the NH_2_ groups in Asn and Gln approximation (2) does not hold, because the rotation around the C−N axis is not uniform. There is a large barrier for the 180° rotation and the rotational motion need not be decoupled from other motions. Experimentally, the two hydrogens are in slow exchange.^[20]^

Order‐parameter target values greater than 0.95 were set to 0.95. The restraining force constant *K* ^sr^ was set to 300 kJmol^−1^, the memory relaxation time to *τ*_sr_=200 ps, and the flat‐bottom parameter of the restraining potential‐energy term to Δ*S* ^2^=0.1, which means a flat bottom of 0.2 width.^[9]^

### MD simulations performed

Four unrestrained MD simulations, starting from the four mentioned X‐ray crystal structures, were performed:

1. MD_2VB1,
2. MD_4LZT,
3. MD_1IEE,
4. MD_1AKI,

each 20 ns long. The average solute temperatures were 311 K and the solvent temperatures 312 K.

Starting from the *2VB1* X‐ray crystal structure, three *S* ^2^‐restraining MD simulations were performed:

1. *MD*_*2VB1*_*Cres*, applying S2CH restraining,
2. *MD*_*2VB1*_*Nres*, applying S2NH restraining,
3. *MD*_*2VB1*_*C+Nres*, applying S2CH and S2NH restraining,

again each 20 ns long. The average solute temperatures were 311 K and the solvent temperatures 312 K. When restraining an order parameter for a bond vector to a target value derived from experiment, the length of the simulation does not play a significant role. It is the restraining force that has to overcome the resistance originating from the particular local protein structure, for example.

### Analysis of atomic trajectories

Trajectory energies and atomic coordinates were stored at 5 ps intervals and used for analysis.^[42]^ *S* ^2^ order parameters were calculated using the ensemble averaging expression^[11]^

(4) $$S_{XY}^{2}=\frac{1}{2}\left\{3\sum_{\alpha =1}^{3}\sum_{\beta =1}^{3}{\left\langle \frac{\mu_{XY\alpha }\left(t\right)\mu_{XY\beta }\left(t\right)}{r_{XY}^{3}\left(t\right)}\right\rangle }_{\tau }^{2}-{\left\langle \frac{1}{r_{XY}^{3}\left(t\right)}\right\rangle }_{\tau }^{2}\right\}{\left(r_{XY}^{eff}\right)}^{6}$$

where *τ* indicates the time‐averaging window, here 1 ns, shorter than the rotational correlation time of 5.7 ns of HEWL in solution,^[22]^

(5) $$\mu {}_{XY1}\equiv \left(x{}_{X}-x{}_{Y}\right)/r{}_{XY},\mu {}_{XY2}\equiv \left(y{}_{X}-y{}_{Y}\right)/r{}_{XY},\mu {}_{XY3}\equiv \left(z{}_{X}-z{}_{Y}\right)/r{}_{XY},$$

are the components of the vector ***r**_XY_*≡***r**_X_*−***r**_Y_* connecting atoms X and Y, and *r_XY_*≡|***r**_XY_*| its length.^[9]^ To obtain a dimensionless quantity the term in curly brackets is multiplied with the 6^th^ power of the effective length (reffXY ) of the vector ***r***_XY_. Because in the present work bond length constraints are used, the length of ***r***_XY_ is essentially constant over time and thus equal to its effective value reffXY .

Before calculating $S_{XY}^{2}$ , the protein trajectory structures are superimposed using the backbone atoms (N, C_α_, C) of residues 3–126 in the fit in order to eliminate the effect of overall rotation of the protein upon the S2XY values. Use of only the backbone atoms of four of the five α‐helices and two β‐strands in HEWL (residues 4–15, 24–36, 41–45, 50–53, 89–99, and 108–115) did not lead to significantly different $S_{XY}^{2}$ values.

In the *S* ^2^ order‐parameter restraining simulations, the *S* ^2^ order parameter is calculated at every time step (2 fs) using an exponential damping factor in the average^[9]^ with a memory relaxation time *τ*_sr_=200 ps, and no rotational fit of the protein structures is carried out, which means that the calculated order parameters in the biased simulation are influenced by the stochastic tumbling of the protein. These *S* ^2^ order‐parameter values will thus differ slightly from the ones calculated from the saved trajectory structures, because in the averages in Equation (4) trajectory structures 5 ps apart are used, the exponential damping factor is not used, the averaging period is 1 ns and a rotational fit of the protein structures is carried out. However, to analyse all trajectories in the same way Equation (4) was used for both the unrestrained and restrained trajectories.

In view of the uncertainty inherent to the derivation of S2XY (exp) values from relaxation experiments and inherent to the calculation of S2XY (MD) values from MD simulation, a deviation of less than 0.2 between simulation and experiment is considered insignificant.

The GROMOS force fields treat aliphatic carbons as united CH, CH_2_ and CH_3_ atoms. So inter‐hydrogen distances involving the aliphatic hydrogen atoms were calculated using virtual atomic positions for CH and pro‐chiral CH_2_ ^[43]^ and pseudo‐atomic positions for CH_3_ ^[44]^ for those hydrogen atoms.^[29]^ The pseudo‐atom NOE distance bound corrections of ref. [44] were used.^[1]^ The set of NOE distance bounds can be found in Table S1 in the Supporting Information, together with values obtained from the seven simulations. The NOE between Trp28 HZ3 and Leu56 HG was reassigned as between Trp28 HZ3 and Leu56 HD* following reassessment of the experimental spectra. Inter‐hydrogen distances were calculated as ⟨*r* ^−3^⟩^−1/3^, that is, using *r* ^−3^ averaging over the trajectory structures, where *r* indicates the actual hydrogen‐hydrogen distance.^[45]^ In view of the uncertainty inherent to the calculation of NOE bounds and *r* ^−3^ averaged distances, deviations from experiment of less than 0.1 nm are considered insignificant.

For the calculation of the side‐chain ^3^ *J*_Hα‐Hβ_ couplings, the Karplus relation[^46^, ^47^] was used with the parameter values *a*=9.5 Hz, *b*=−1.6 Hz and *c*=1.8 Hz.^[48]^ In view of the various factors contributing to an uncertainty of about 2 Hz inherent to the Karplus relation linking structure and ^3^ *J* couplings, a deviation of less than 2 Hz between ^3^ *J*_Hα‐Hβ_ coupling values calculated from MD trajectory structures and ^3^ *J*_Hα‐Hβ_ coupling values derived from experiment is considered insignificant.

Atom‐positional root‐mean‐square differences RMSD(*t*) between trajectory structures and the X‐ray crystal structures and atom‐positional root‐mean‐square fluctuations (RMSF), i. e. around their average positions, in the MD trajectories were calculated after superimposing the backbone atoms (N, C_α_, C) of residues 3–126 to eliminate the contribution of overall translation and rotation of the protein.

The secondary structure assignment was done with the program DSSP, based on the Kabsch‐Sander rules.^[49]^

Hydrogen bonds were identified according to a geometric criterion: a hydrogen bond was assumed to exist if the hydrogen‐acceptor distance was smaller than 0.25 nm and the donor‐hydrogen‐acceptor angle was larger than 135°.

## 2 Results and Discussion

### 2.1 Comparison of *S* ^2^ order‐parameter values calculated from unrestrained MD trajectories with NMR derived values

Table 1 lists 51 S2CH values derived from relaxation measurements and from four unrestrained MD simulations starting from four X‐ray crystal structures. The mean of the four MD values and the root‐mean‐square deviation (RMSD) from it are also presented. As order parameters in an MD simulation can only be equal to 1 if there is no motion at all, order‐parameter target values larger than 0.95 were set to 0.95. Deviations from the experimentally derived values of more than 0.2 are denoted in italics. The mean values of the MD simulations show 18 deviations larger than 0.2, 12 are smaller and six are larger than the experimentally derived value. Some large (>0.8) experimentally derived values, for example, for Met12 CE (0.812), Leu25 CD1 (1.0), Val29 CG1 (0.871), Thr69 CG2 (0.98), Leu84 CD1 (1.0) and CD2 (0.879), Thr89 CG2 (1.0), are not reproduced within 0.2 in any of the four MD simulations. Some small (<0.4) experimentally derived values, for example, for Thr43 CG2 (0.361), Thr47 CG2 (0.327), Ile55 CD (0.323), and Ile58 CD (0.160), are also not reproduced within 0.2 in any of the four MD simulations. Large order parameters are expected for side chains buried inside the protein, while small order parameters are expected for side chains at the protein surface. Yet, this seems not always true. Val99 has in the 2VB1 X‐ray structure a solvent accessible area of only 7 % and its side chain is surrounded by the side chains of Tyr20, Trp28, Ile98, and Tyr108. This leads to simulated S2CH values of about 0.71 to be compared to the experimentally derived value of 0.5. Thr89 has in the 2VB1 X‐ray structure a solvent accessible area of 76 %. This leads to simulated S2CH values of about 0.68 to be compared to the experimentally derived value of 1.0. A larger variation (RMSD≥0.12) of S2CH values between the four MD simulations is observed for Met12 CE, Leu75 CD1, Ile78 CG2, Val99 CG1 and CG2, and Met105 CE.

Table 2 lists the 11 S2NH values for Trp (NE1‐HE1) and Arg (NE‐HE) side chains derived from relaxation measurements and from four unrestrained MD simulations starting from four X‐ray crystal structures. Three simulations show only one deviation from the experimentally derived value larger than 0.2. Both, large and small values are well reproduced.

Table 3 lists the 17 S2NH values for Asn (ND2‐HD21, ‐HD22) and Gln (NE2‐HE21, ‐HE22) side chains derived from relaxation measurements and from four unrestrained MD simulations starting from four X‐ray crystal structures. The simulations show one or two deviations from the experimentally derived value larger than 0.2, four simulated values, for Asn65 and 74, smaller than the experimentally derived values, and two simulated values, for Asn103 and 113, larger than the experimentally derived ones.

Table 4 shows the occurrence (%) of hydrogen bonds involving the side chains of Arg, Asn, Gln and Trp residues in the four X‐ray structures and in the four unrestrained MD simulations starting from the four respective X‐ray structures. The four hydrogen bonds present in all four X‐ray structures are also observed in the MD simulations, but with widely different occurrences (0–85 %). For the seven hydrogen bonds observed in only three of the four X‐ray structures the occurrences vary from 0 to 99 %. Considering the different thermodynamic conditions under which the X‐ray diffraction of the different crystals was measured and the pH of the NMR measurement in aqueous solution, the observed differences are not surprising.

**Table 4:** Occurrence (%) of hydrogen bonds (52) involving the side chains of Arg, Asn, Gln and Trp residues in four X‐ray structures and in the four unrestrained MD simulations starting from the four X‐ray structures. Only hydrogen bonds present in one of the X‐ray structures or with a population of at least 20 % in any of the restrained or unrestrained MD simulations are included. Only hydrogen bond populations of 1 % or greater are shown.

Hydrogen bond	X‐ray structure				MD simulation			
Donor‐acceptor	*2VB1*	*4LZT*	*1IEE*	*1AKI*	*2VB1*	*4LZT*	*1IEE*	*1AKI*
Arg5 NE‐HE‐Trp123 O					15	21	10	1
Arg5 NH1/2‐HH1/2‐Arg125 O*	100	100	100	100	9	3	5	3
Arg5 NH1‐HH12‐Trp123 O	100	100	100	100	22	17	24	13
Asn19 ND2‐HD22‐Asp18 OD1/2*					11	10	10	19
Asn27 ND2‐HD22‐Trp111 O					–	2	–	–
Asn27 ND2‐HD22‐Ser24 O				100	–	13	1	1
Asn27 ND2‐HD22‐Ser24 OG					–	11	2	–
Asn27 ND2‐HD22‐Cys115 O					–	19	1	78
Trp28 NE1‐HE1‐Leu17 O					–	2	–	29
Trp28 NE1‐HE1‐Tyr23 O		100			–	32	8	–
Gln41 NE2‐HE22‐Leu84 O				100	2	3	1	–
Asn44 ND2‐HD22‐Asp52 OD1/2*	100	100			61	43	34	34
Asn44 ND2‐HD22‐Gln57 OE1			100		14	2	27	5
Arg45 NH1‐HH12‐Gly49 O			100		8	2	–	4
Asn46 ND2‐HD21‐Ala107 O					56	–	4	–
Asn46 ND2‐HD1/2‐Asp52 OD1/2*	100	100	100		75	40	61	55
Asn46 ND2‐HD22‐Ser50 OG	100	100	100		1	24	35	10
Asn46 ND2‐HD22‐Ser50 O					2	24	36	10
Gln57 NE2‐HE21‐Glu35 OE1					1	–	–	36
Gln57 NE2‐HE21‐Ala42 O					66	7	21	–
Gln57 NE2‐HE22‐Ser36 OG					6	46	2	23
Gln57 NE2‐HE22‐Gly54 O	100	100	100	100	85	16	49	–
Asn59 ND2‐HD21‐Ser50 OG	100	100		100	99	94	79	96
Asn59 ND2‐HD1/2‐Asp52 OD1/2*	100	100		100	70	54	50	50
Arg61 NH2‐HH22‐Asp48 OD2	100	100		100	1	–	–	–
Arg61 NH2‐HH21‐Asp48 O		100			–	–	1	–
Arg61 NE‐HE‐Thr69 OG1					6	16	13	28
Trp63 NE1‐HE1‐Asn106 OD1					11	–	28	–
Asn65 ND2‐HD22‐Asn74 OD1					52	15	35	14
Arg68 NH2‐HH22‐Thr51 OG1			100		–	2	–	–
Arg73 NH1‐HH12‐Arg61 O					13	11	28	13
Asn74 ND2‐HD21‐Asn77 O					–	3	–	22
Asn103 ND2‐HD22‐Ile98 O	100	100			–	–	–	–
Asn103 ND2‐HD22‐Asp101 OD1/2*					42	3	23	–
Asn106 ND2‐HD22‐Gly102 O					–	21	–	–
Asn106 ND2‐HD22‐Asn103 O			100	100	3	–	–	3
Asn106 ND2‐HD22‐Asn103 OD1					22	–	4	1
Trp108 NE1‐HE1‐Leu56 O			100		91	59	94	45
Trp111 NE1‐HE1‐Asn27 OD1	100	100	100	100	1	16	2	1
Trp111 NE1‐HE1‐Asn27 O					–	–	–	–
Arg112 NE‐HE‐Asn106 O					20	8	1	27
Arg112 NH1‐HH12‐Asn106 O	100	100		100	19	28	2	4
Asn113 ND2‐HD21‐Val109 O					2	–	66	1
Arg114 NH1‐HH12‐Glu35 OE1					–	25	–	–
Arg114 NE‐HE‐Asn113 OD1					9	3	26	26
Gln121 NE2‐HE22‐Asp119 OD1/2*					22	10	34	27
Trp123 NE1‐HE1‐Gly117 O					14	–	20	49
Trp123 NE1‐HE1‐Thr118 OG1					14	48	–	3
Arg125 NE‐HE‐Gln121 OE1	100				2	5	–	1
Arg125 NH2‐HH22‐Asp119 OD2	100	100		100	–	–	–	–
Arg125 NH2‐HH22‐Gln121 OE1	100	100			1	4	1	1
Arg125 NH1‐HH12‐Ala122 O			100		–	–	1	1

* Some hydrogen bonds involving aspartic acid side chains are present in the simulations with either OD1 or OD2 acting as the acceptor. In these cases (marked OD1/2) the highest population of the hydrogen bond involving either OD1 or OD2 is listed. Similarly for hydrogen bonds involving asparagine NH_2_ groups in some cases (marked ND2‐HD1/2) the highest population of a hydrogen bond where the donor is either ND2‐HD21 or ND2‐HD22 is listed while for arginine NH2 groups in some cases (marked NH1/2‐HH1/2) the highest population of a hydrogen bond where the donor is either NH1‐HH11, NH1‐HH12, NH2‐HH21 or NH2‐HH22 is listed.

**Figure 2:**
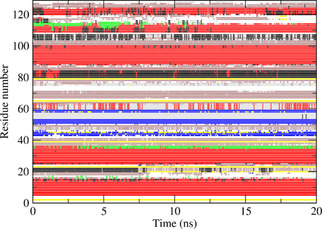
Secondary structure elements^[49]^ as a function of time calculated for the unrestrained MD simulation *MD*_*2VB1* starting from the 2VB1 X‐ray structure. Red: α‐helix; green: π‐helix; black: 3_10_‐helix; blue: β‐strand; yellow: β‐bridge; brown: bend; grey: turn.

Figures 2 and S1–S3 show the secondary structure elements^[49]^ of HEWL as a function of time calculated for the four unrestrained MD simulations. Five α‐helices (red; residues 4–15, 24–36, 89–99, 108–115 and 121–125) and three β‐strands (blue; residues 41–45, 50–53 and 58–59) are largely maintained, but the α‐helix at residues 108–115 turns into two β‐bridges (yellow) after 3 ns in the *MD*_*1IEE* simulation (Figure S2). All four simulations show a helix of alternating α‐helical (red) and 3_10_‐helical (black) character at residues 80–85. At residues 21–24, simulation *MD*_*1AKI* (Figure S3) shows a 3_10_‐helix, which is lost after about 6 ns in the *MD*_*2VB1* (Figure 2) and *MD*_*1IEE* (Figure S2) simulations, and within 1 ns in the *MD*_*4LZT* simulation (Figure S1).

**Figure 3:**
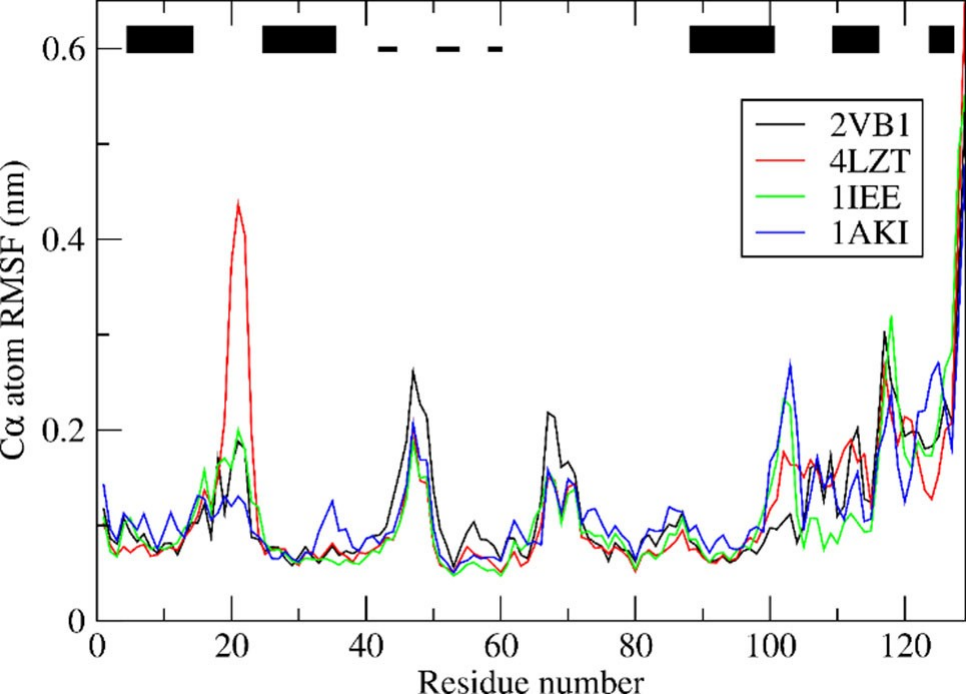
Backbone Cα atom‐positional root‐mean‐square fluctuations (RMSF) as function of residue sequence number in the four unrestrained MD simulations *MD*_*2VB1* (black), *MD*_*4LZT* (red), *MD*_*1IEE* (green) and *MD*_*1AKI* (blue) starting from the respective four X‐ray structures. The trajectory structures are translationally and rotationally superimposed using the backbone atoms (N, Cα, C) of residues 3–126. The black bars at the top indicate secondary structure elements of HEWL (thick bars: α‐helix; thin bars, β‐strand).

Figure 3 shows the backbone C_α_ atom‐positional root‐mean‐square fluctuations (RMSF) as function of residue sequence number in the four unrestrained MD simulations *MD*_*2VB1* (black), *MD*_*4LZT* (red), *MD*_*1IEE* (green) and *MD*_*1AKI* (blue) starting from the respective four X‐ray structures. Apart from the residues beyond residue sequence number 100 at the C‐terminal part of the polypeptide chain, the motional patterns in the four simulations are rather similar, except for residues 21–24 in the *MD*_*4LZT* (red) simulation that become very mobile, their initial 3_10_‐helical character being lost.

### 2.2 Comparison of *S* ^2^ order‐parameter values calculated from *S* ^2^ order‐parameter restraining MD trajectories with NMR derived values

Table 5 lists the 51 S2CH values derived from relaxation measurements and from the unrestrained and order‐parameter restrained MD simulations starting from the *2VB1* X‐ray crystal structure using three different sets of *S* ^2^ order‐parameter restraints. The unrestrained MD simulation shows 22 *S* ^2^ order‐parameter values (in italics) that deviate more than 0.2 from the experimentally derived values (in 17 residues: 2, 12, 25, 29, 43, 47, 51, 55, 58, 69, 83, 84, 88, 89, 95, 99 and 129). *S* ^2^ order‐parameter restraining towards the set *Cres* of 51 target S2CH (exp) values leads, as expected, to good agreement between simulation and experiment for the 51 S2CH order parameters. Only three deviations larger than 0.2 are observed, for Ala95 CB and for Leu129 CD1 and CD2. Tables 6 and 7 show that restraining towards the set *Cres* of 51 target S2CH (exp) values very slightly worsens the agreement between simulation and experiment for the S2NH (exp) values. Worsening by more than 0.1 is observed for Trp108, Trp111, and Arg112 (Table 6), and for Gln57 HE21 and Asn65 HD22 (Table 7). Yet, also some improvement by 0.1 of the agreement between simulation and experiment for the S2NH values is observed, for example, for Asn44 HD22, Asn46 HD22 and Asn103 HD22 (Table 7).

**Table 5:** S2CH values (51) derived from relaxation measurements and from the unrestrained and order‐parameter restrained MD simulations starting from the 2VB1 X‐ray crystal structure. Order‐parameter target values larger than 0.95 were set to 0.95 (second column between brackets). Values differing more than 0.2 from the experimental value (0.95 in case the experimental value is 1) are denoted using italics.

Residue and methyl group	Experimental value^[21]^	Unrestrained MD	Order‐parameter restrained MD		
		*2VB1*	*2VB1*_*Cres*	*2VB1*_*Nres*	*2VB1*_*C+Nres*
Val2 CG2	0.598	*0.39*	0.42	0.46	0.44
Leu8 CD1	0.767	0.58	0.64	0.65	0.67
Leu8 CD2	0.803	0.63	0.72	0.66	0.71
Ala9 CB	1.0 (0.95)	0.93	0.93	0.93	0.93
Ala10 CB	0.901	0.91	0.92	0.92	0.92
Ala11 CB	0.861	0.91	0.92	0.92	0.92
Met12 CE	0.812	*0.33*	0.90	*0.46*	0.87
Leu17 CD1	0.630	0.46	0.51	0.78	0.56
Leu17 CD2	0.632	0.49	0.58	0.77	0.61
Leu25 CD1	1.0 (0.95)	*0.40*	0.81	*0.64*	0.81
Leu25 CD2	0.609	0.42	0.69	0.69	0.75
Val29 CG1	0.871	*0.57*	0.85	*0.62*	0.82
Val29 CG2	0.791	*0.57*	0.84	0.61	0.81
Ala31 CB	0.98 (0.95)	0.94	0.95	0.93	0.94
Thr43 CG2	0.361	*0.68*	0.43	*0.74*	0.35
Thr47 CG2	0.327	*0.73*	0.40	*0.73*	0.42
Thr51 CG2	0.778	*0.49*	0.85	*0.54*	0.79
Ile55 CG2	0.739	*0.49*	0.63	*0.53*	0.69
Ile55 CD	0.323	*0.55*	0.41	*0.56*	0.40
Leu56 CD1	0.734	0.79	0.63	0.56	0.74
Leu56 CD2	0.681	0.75	0.66	0.53	0.68
Ile58 CG2	1.0 (0.95)	0.84	0.87	*0.72*	0.88
Ile58 CD	0.160	*0.81*	0.20	*0.78*	0.22
Thr69 CG2	0.98 (0.95)	*0.72*	0.89	0.84	0.88
Leu75 CD1	0.590	0.62	0.69	0.52	0.56
Ile78 CG2	0.810	0.85	0.83	*0.59*	0.80
Ile78 CD	0.416	0.43	0.45	0.29	0.40
Leu83 CD1	0.884	*0.68*	0.79	*0.35*	0.77
Leu83 CD2	0.783	0.66	0.72	*0.41*	0.71
Leu84 CD1	1.0 (0.95)	*0.46*	0.86	*0.60*	0.86
Leu84 CD2	0.879	*0.45*	0.83	*0.58*	0.84
Ile88 CG2	0.697	0.55	0.66	0.81	0.66
Ile88 CD	0.722	*0.27*	0.64	*0.49*	0.58
Thr89 CG2	1.0 (0.95)	*0.71*	0.87	*0.69*	0.85
Ala90 CB	0.919	0.91	0.92	0.92	0.91
Val92 CG1	0.764	0.63	0.80	0.89	0.86
Val92 CG2	0.707	0.61	0.79	0.87	0.83
Ala95 CB	0.680	*0.94*	*0.91*	*0.94*	*0.91*
Ile98 CG2	0.740	0.90	0.87	0.73	0.83
Ile98 CD	0.815	0.89	0.86	0.82	0.85
Val99 CG1	0.487	*0.85*	0.38	0.53	0.45
Val99 CG2	0.517	*0.85*	0.37	0.52	0.42
Met105 CE	0.630	0.80	0.76	*0.37*	0.79
Ala107 CB	0.832	0.88	0.87	0.82	0.85
Val109 CG2	0.354	0.36	0.22	0.32	0.25
Val120 CG1	0.660	0.69	0.52	0.52	0.57
Ala122 CB	0.879	0.78	0.86	0.81	0.83
Ile124 CG2	0.753	0.75	0.79	*0.50*	0.70
Ile124 CD	0.351	0.48	0.49	0.44	0.34
Leu129 CD1	0.525	*0.12*	*0.27*	*0.12*	0.33
Leu129 CD2	0.507	*0.11*	*0.27*	*0.10*	0.31

**Table 6:** S2NH values (11) for Trp (NE1‐HE1) and Arg (NE‐HE) side chains derived from relaxation measurements and from the unrestrained and order‐parameter restrained MD simulations starting from the *2VB1* X‐ray crystal structure.

Residue	Experimental value^[20]^	Unrestrained MD	Order‐parameter restrained MD		
		*2VB1*	*2VB1*_*Cres*	*2VB1*_*Nres*	*2VB1*_*C+Nres*
Trp28	0.90	0.88	0.84	0.85	0.89
Trp62	0.41	*0.73*	*0.72*	0.48	0.54
Trp63	0.88	0.83	0.81	0.87	0.85
Trp108	0.87	0.87	0.70	0.81	0.83
Trp111	0.88	0.83	*0.59*	0.81	0.78
Trp123	0.85	0.70	0.67	0.82	0.78
Arg61	0.28	0.22	0.30	0.26	0.29
Arg73	0.12	0.24	0.21	0.13	0.13
Arg112	0.31	0.28	0.13	0.23	0.22
Arg114	0.27	0.13	0.18	0.13	0.19
Arg125	0.05	0.12	0.13	0.11	0.10

**Table 7:** S2NH values (17) for Asn (ND2‐HD21, ‐HD22) and Gln (NE2‐HE21, ‐HE22) side chains derived from relaxation measurements and from the unrestrained and order‐parameter restrained MD simulations starting from the 2VB1 X‐ray crystal structure. The experimental values correspond to either HD/E21 or HD/E22.^[20]^ The assignment in the second column is based on the best agreement with the values of the *MD*_*2VB1* simulation (third column). The N−H vectors used as restraint are indicated by * . Values differing more than 0.2 from the experimental value are denoted using italics.

Residue	Experimental value^[20]^	Unrestrained MD	Order‐parameter restrained MD		
		*2VB1*	*2VB1*_*Cres*	*2VB1*_*Nres*	*2VB1*_*C+Nres*
Asn19 HD21*	0.43	0.49	0.56	0.43	0.39
Asn19 HD22		0.24	0.45	0.28	0.25
Asn27 HD21		0.86	0.79	0.76	0.82
Asn27 HD22*	0.72	0.82	0.57	0.71	0.66
Asn37 HD21*	0.51	0.37	0.32	0.50	0.51
Asn37 HD22		0.21	0.20	0.21	0.20
Asn39 HD21*	0.74	0.80	0.77	0.80	0.79
Asn39 HD22		0.61	0.57	0.59	0.57
Gln41 HE21		0.31	0.45	0.42	0.35
Gln41 HE22*	0.19	0.21	0.22	0.21	0.20
Asn44 HD21		0.75	0.66	0.63	0.65
Asn44 HD22*	0.51	0.71	0.56	0.57	0.58
Asn46 HD21		0.85	0.85	0.84	0.75
Asn46 HD22*	0.62	0.82	0.52	0.69	0.56
Gln57 HE21*	0.82	0.79	*0.56*	0.80	0.76
Gln57 HE22		0.76	0.23	0.66	0.48
Asn59 HD21		0.92	0.89	0.91	0.91
Asn59 HD22*	0.78	0.90	0.87	0.88	0.87
Asn65 HD21		0.76	0.78	0.50	0.55
Asn65 HD22*	0.57	0.42	*0.30*	0.43	0.43
Asn74 HD21*	0.74	0.66	0.65	0.71	0.75
Asn74 HD22		0.41	0.38	0.30	0.46
Asn77 HD21		0.54	0.50	0.45	0.47
Asn77 HD22*	0.24	0.31	0.28	0.24	0.24
Asn93 HD21*	0.59	0.53	0.63	0.61	0.58
Asn93 HD22		0.34	0.39	0.30	0.29
Asn103 HD21		0.72	0.33	0.31	0.29
Asn103 HD22*	0.26	*0.61*	0.21	0.17	0.17
Asn106 HD21*	0.58	0.68	0.40	0.48	0.47
Asn106 HD22		0.46	0.23	0.21	0.20
Asn113 HD21*	0.47	0.40	0.41	0.39	0.41
Asn113 HD22		0.21	0.24	0.18	0.21
Gln121 HE21*	0.36	0.34	0.37	0.28	0.31
Gln121 HE22		0.18	0.17	0.15	0.14

Tables 6 and 7 show that in the unrestrained MD simulation only two S2NH order‐parameter values (in italics) deviate more than 0.2 from the experimentally derived values, for Trp62 (Table 6) and for Asn103 HD22 (Table 7). *S* ^2^ order‐parameter restraining towards the set *Nres* of 28 target S2NH (exp) values leads, as expected, to good agreement between simulation and experiment for the 28 S2NH order parameters. No deviations larger than 0.2 are observed.

*S* ^2^ order‐parameter restraining towards the set *C+Nres* of 79 target S2CH (exp) and S2NH (exp) values leads, as expected, to good agreement between simulation and experiment for 78 *S* ^2^ order parameters (Figure S4), only one deviation larger than 0.2 is observed, for the S2CH value of Ala95 CB (Table 5). The *S* ^2^ order‐parameter restraining is not able to reduce the S2CH (MD) value from 0.94 in the unrestrained simulation to the target S2CH (exp) value of 0.68. Enhancing the mobility of the CA–CB vector that is close to the polypeptide backbone and in a residue that is in the centre of a helix seems impossible with the parameters applied here.

Table 5 shows that for the 51 S2CH order parameters restraining towards the set *Nres* of 28 target S2NH (exp) values yields 21 deviations larger than 0.2. Restraining towards the S2NH (exp) values does not improve the overall agreement between simulation and experiment for the S2CH values. Yet, for some S2CH order parameters the agreement improves (Met12 CE, Leu25 CD1 and CD2, Thr69 CG2, Leu84 CD1 and CD2, Ile88 CD2, Ile98 CG2, Val99 CG1 and CG2) by more than 0.1, and for some S2CH order parameters the agreement worsens (Leu56 CD1 and CD2, Ile58 CG2, Ile78 CG2 and CD, Leu83 CD1 and CD2, Met105 CE, Val120 CG1 and Ile124 CG2) by more than 0.1.

Table 8 shows the occurrence (%) of hydrogen bonds involving the side chains of Arg, Asn, Gln and Trp residues in the *MD*_*2VB1* unrestrained MD simulation and in the three *S* ^2^ order‐parameter restraining MD simulations starting from the *2VB1* X‐ray structure. The unrestrained simulation shows nine hydrogen bonds with an occurrence larger than 50 %. *S* ^2^ order‐parameter restraining MD simulation reduces this number to 5, 3 and 3 for the three *S* ^2^ order‐parameter restraining MD simulations using the *Cres*, *Nres* or *C+Nres* sets of restraints, respectively. All but one of the occurrences of the mentioned 9 hydrogen bonds are reduced by the *S* ^2^ order‐parameter restraining. In contrast, only one hydrogen‐bond occurrence is raised above 50 % by restraining, the hydrogen bond Asn46 ND2‐HD22–Ser50 O, from 2 % to 51, 27 and 17 %, respectively. Of the 52 hydrogen bonds listed (i. e., observed in either the four X‐ray structures or for at least 20 % in the seven MD simulations), 38 are observed in the unrestrained simulation, 39, 37 and 42 are observed in the three *S* ^2^ order‐parameter restraining MD simulations using the *Cres*, *Nres* or *C+Nres* sets of restraints, respectively.

**Table 8:** Occurrence (%) of hydrogen bonds (52) involving the side chains of Arg, Asn, Gln and Trp residues in the *MD*_*2VB1* unrestrained MD simulation and in the three *S* ^2^ order‐parameter restraining MD simulations starting from the 2VB1 X‐ray structure. Only hydrogen bonds present in one of the X‐ray structures or with a population of at least 20 % in any of the restrained or unrestrained MD simulations are included. Only hydrogen bond populations of 1 % or greater are shown.

Hydrogen bond	MD simulation			
	Unrestrained	Order‐parameter restrained		
Donor‐acceptor	*2VB1*	*2VB1*_*Cres*	*2VB1*_*Nres*	*2VB1*_*C+Nres*
Arg5 NE‐HE‐Trp123 O	15	6	14	9
Arg5 NH1/2‐HH1/2‐Arg125 O*	9	4	7	8
Arg5 NH1‐HH12‐Trp123 O	22	27	10	12
Asn19 ND2‐HD22‐Asp18 OD1/2*	11	35	30	24
Asn27 ND2‐HD22‐Trp111 O	–	–	26	4
Asn27 ND2‐HD22‐Ser24 O	–	2	9	1
Asn27 ND2‐HD22‐Ser24 OG	–	3	21	–
Asn27 ND2‐HD22‐Cys115 O	–	24	7	6
Trp28 NE1‐HE1‐Leu17 O	–	30	30	7
Trp28 NE1‐HE1‐Tyr23 O	–	15	23	4
Gln41 NE2‐HE22‐Leu84 O	2	2	2	3
Asn44 ND2‐HD22‐Asp52 OD1/2*	61	41	30	33
Asn44 ND2‐HD22‐Gln57 OE1	14	3	6	3
Arg45 NH1‐HH12‐Gly49 O	8	2	4	3
Asn46 ND2‐HD21‐Ala107 O	56	–	–	–
Asn46 ND2‐HD1/2‐Asp52 OD1/2*	75	68	41	40
Asn46 ND2‐HD22‐Ser50 OG	1	25	23	23
Asn46 ND2‐HD22‐Ser50 O	2	51	27	17
Gln57 NE2‐HE21‐Glu35 OE1	1	–	–	1
Gln57 NE2‐HE21‐Ala42 O	66	5	24	4
Gln57 NE2‐HE22‐Ser36 OG	6	9	4	17
Gln57 NE2‐HE22‐Gly54 O	85	18	71	8
Asn59 ND2‐HD21‐Ser50 OG	99	84	95	93
Asn59 ND2‐HD1/2‐Ser52 OD1/2*	70	60	52	52
Arg61 NH2‐HH22‐Asp48 OD2	1	–	–	9
Arg61 NH2‐HH21‐Asp48 O	–	–	–	–
Arg61 NE‐HE‐Thr69 OG1	6	–	–	–
Trp63 NE1‐HE1‐Asn106 OD1	11	–	–	–
Asn65 ND2‐HD22‐Asn74 OD1	52	56	3	2
Arg68 NH2‐HH22‐Thr51 OG1	–	2	–	3
Arg73 NH1‐HH12‐Arg61 O	13	10	6	11
Asn74 ND2‐HD21‐Asn77 O	1	1	7	8
Asn103 ND2‐HD22‐Ile98 O	–	–	–	–
Asn103 ND2‐HD22‐Asp101 OD1/2	42	5	1	4
Asn106 ND2‐HD22‐Gly102 O	–	5	1	1
Asn106 ND2‐HD22‐Asn103 O	3	3	–	1
Asn106 ND2‐HD22‐Asn103 OD1	22	–	–	1
Trp108 NE1‐HE1‐Leu56 O	91	9	24	54
Trp111 NE1‐HE1‐Asn27 OD1	1	17	22	1
Trp111 NE1‐HE1‐Asn27 O	–	–	23	19
Arg112 NE‐HE‐Asn106 O	20	3	4	4
Arg112 NH1‐HH12‐Asn106 O	19	9	16	6
Asn113 ND2‐HD21‐Val109 O	2	–	8	–
Arg114 NH1‐HH12‐Glu35 OE1	–	2	–	11
Arg114 NE‐H‐Asn113 OD1	9	2	–	–
Gln121 NE2‐HE22‐Asp119 OD1/2*	22	12	20	16
Trp123 NE1‐HE1‐Gly117 O	14	1	–	1
Trp123 NE1‐HE1‐Thr118 OG1	14	5	45	11
Arg125 NE‐HE‐Gln121 OE1	2	4	5	4
Arg125 NH2‐HH22‐Asp119 OD2	–	–	–	–
Arg125 NH2‐HH22‐Gln121 OE1	1	3	3	3
Arg125 NH1‐HH12‐Ala122 O	–	–	–	–

* Some hydrogen bonds involving aspartic acid side chains are present in the simulations with either OD1 or OD2 acting as the acceptor. In these cases (marked OD1/2) the highest population of the hydrogen bond involving either OD1 or OD2 is listed. Similarly for hydrogen bonds involving asparagine NH_2_ groups in some cases (marked ND2‐HD1/2) the highest population of a hydrogen bond where the donor is either ND2‐HD21 or ND2‐HD22 is listed while for arginine NH2 groups in some cases (marked NH1/2‐HH1/2) the highest population of a hydrogen bond where the donor is either NH1‐HH11, NH1‐HH12, NH2‐HH21 or NH2‐HH22 is listed.

There are a number of examples where the *MD*_*2VB1* unrestrained simulation yields a *S* ^2^ value larger than experiment. When restraining reduces the *S* ^2^ value, a reduction or change in the populations of hydrogen bonds involving that side chain is observed. For example, Asn44 (experimental S2NH value 0.51 compared to 0.71 and 0.58 in the *MD*_*2VB1* and *MD*_*2VB1*_*C+Nres* simulations, respectively, Table 7) shows a reduction in the populations of hydrogen bonds to the side chains of Asp52 and Gln57 (Table 8) and Asn46 (experimental S2NH value 0.62 compared to 0.82 and 0.56 in the *MD*_*2VB1* and *MD*_*2VB1*_*C+Nres* simulations, respectively) shows a reduction in the population of the hydrogen bond to the side chain of Asp52, but an increase in the population of hydrogen bonds to both the main chain and side chain oxygens of Ser50 in the restrained simulations. Similarly, for Asn103 (experimental S2NH value 0.26 compared to 0.61 and 0.17 in the *MD*_*2VB1* and *MD*_*2VB1*_*C+Nres* simulations, respectively), the hydrogen bond to the side chain of Asp101 is almost completely lost in the restrained simulations.

**Figure 4:**
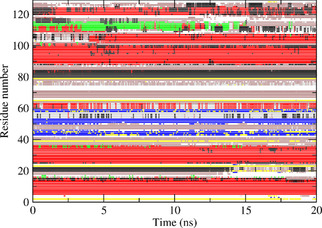
Secondary structure elements^[49]^ as a function of time calculated for the S2CH order‐parameter restraining MD simulation *MD*_*2VB1*_*Cres* starting from the 2VB1 X‐ray structure. Red: α‐helix; green: π‐helix; black: 3_10_‐helix; blue: β‐strand; yellow: β‐bridge; brown: bend; grey: turn.

**Figure 5:**
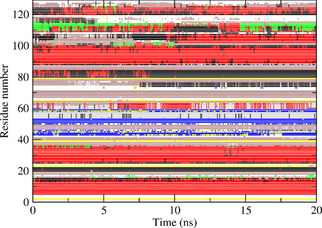
Secondary structure elements^[49]^ as a function of time calculated for the S2NH order‐parameter restraining MD simulation *MD*_*2VB1*_*Nres* starting from the *2VB1* X‐ray structure. Red: α‐helix; green: π‐helix; black: 3_10_‐helix; blue: β‐strand; yellow: β‐bridge; brown: bend; grey: turn.

**Figure 6:**
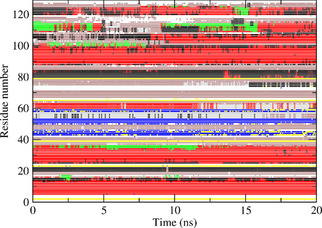
Secondary structure elements^[49]^ as a function of time calculated for the S2CH and S2NH order‐parameter restraining MD simulation *MD*_*2VB1*_*N+Cres* starting from the *2VB1* X‐ray structure. Red: α‐helix; green: π‐helix; black: 3_10_‐helix; blue: β‐strand; yellow: β‐bridge; brown: bend; grey: turn.

Figures 4 to 6 show the secondary structure elements^[49]^ of HEWL as a function of time calculated for the three *S* ^2^ order‐parameter restraining MD simulations starting from the *2VB1* X‐ray structure. Compared to the *MD*_*2VB1* unrestrained simulation (Figure 2), S2CH order‐parameter restraining towards the S2CH (exp) values of set *Cres* induces some changes in secondary structure (Figure 4). Residues 19–21 become 3_10_‐helical, the second α‐helix (residues 24–36) is slightly more stable at its C‐terminal end, the helix of alternating α‐helical and 3_10_‐helical character at residues 80–85 gains 3_10_‐helical character, residues 103–108 become α‐helical after 4 ns, and the α‐helix 108–115 gains π‐helical character. Generally, the 3_10_‐helical character is increased. S2NH order‐parameter restraining towards the S2NH (exp) values of set *Nres* shows similar changes in the secondary structure (Figure 5). The same observation holds for the *MD*_*2VB1*_*C+Nres* simulation (Figure 6).

**Figure 7:**
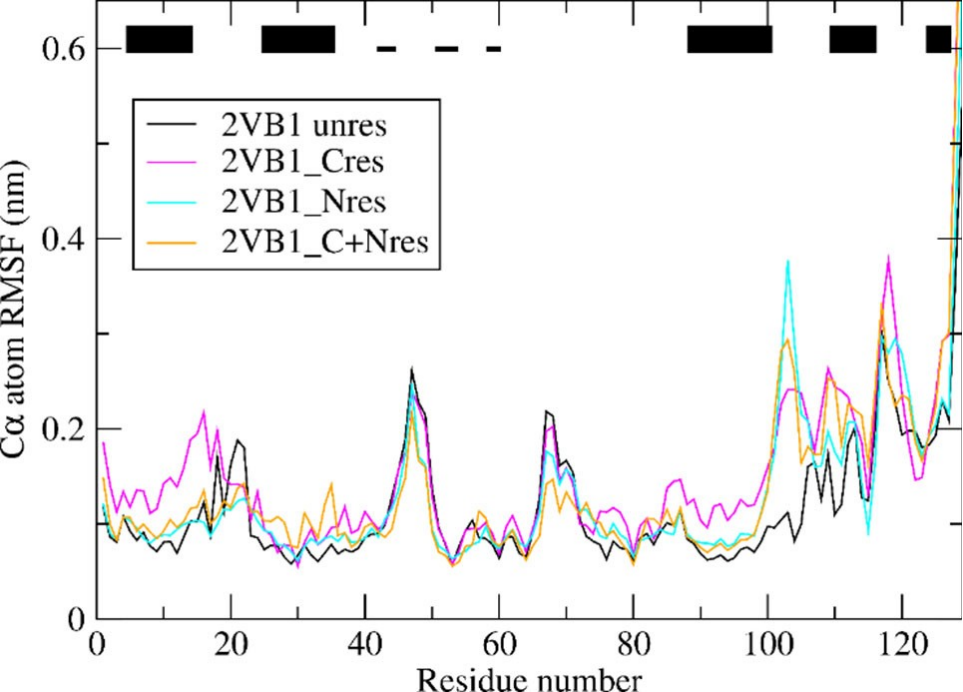
Backbone C_α_ atom‐positional root‐mean‐square fluctuations (RMSF) as function of residue sequence number for the unrestrained MD simulation *MD*_*2VB1* (black) and for the three *S* ^2^ order‐parameter restraining MD simulations *MD*_*2VB1*_*Cres* (magenta), *MD*_*2VB1*_*Nres* (cyan) and *MD*_*2VB1*_*C+Nres* (orange) all starting from the *2VB1* X‐ray structure. The trajectory structures are translationally and rotationally superimposed using the backbone atoms (N, C_α_, C) of residues 3–126. The black bars at the top indicate secondary structure elements of HEWL (thick bars: α‐helix; thin bars, β‐strand).

Figure 7 shows the backbone C_α_ atom‐positional root‐mean‐square fluctuations (RMSF) as function of residue sequence number in the unrestrained MD simulation *MD*_*2VB1* (black) and in the *S* ^2^ order‐parameter restraining simulations *MD*_*2VB1*_*Cres* (magenta), *MD*_*2VB1*_*Nres* (cyan) and *MD*_*2VB1*_*C+Nres* (orange) all starting from the *2VB1* X‐ray structure. S2CH order‐parameter restraining induces mobility for residues 1–17, residues 85–105 and residues 109–112. Restraining S2NH order parameters shows increased mobility for residues 100–105. Restraining to both sets of order parameters shows increased mobility for residues 100–104 and 109–110.

### 2.3 Discussion

Table 9 summarises the deviations of S2CH (MD) values from S2CH (exp) values for the 51 S2CH order parameters in the seven MD simulations. Of course, S2CH order‐parameter restraining reduces the number of larger deviations. S2NH order‐parameter restraining increases the number of deviations larger than 0.1 from 33 in the unrestrained *MD*_*2VB1* simulation to 35 in the *MD*_*2VB1*_*Nres* simulation. *S* ^2^ order‐parameter restraining to all 79 experimentally derived *S* ^2^ order‐parameter values yields even less deviations larger than 0.1 (12) than restraining only to the 51 S2CH order‐parameter values (16).

**Table 9:** Number of deviations, |*S* ^2^(exp) ‐ *S* ^2^(MD)|, for the 51 S2CH values in the seven MD simulations.

Simulation	Size of *S* ^2^ deviation					
	0.05–0.1	0.1–0.2	0.2–0.3	0.3–0.4	0.4–0.5	>0.5
MD_2VB1	7	12	9	6	5	1
MD_4LZT	6	11	8	7	1	1
MD_1IEE	9	11	9	5	1	3
MD_1AKI	7	6	16	8	0	2
MD_2VB1_Cres	19	13	3	0	0	0
MD_2VB1_Nres	6	13	11	7	2	2
MD_2VB1_C+Nres	17	11	1	0	0	0

Tables 10 and 11 summarise the deviations of S2NH (MD) values from S2NH (exp) values for the 28 S2NH order parameters in the seven MD simulations. Of course, S2NH order‐parameter restraining marginally improves the agreement with experiment. S2CH order‐parameter restraining does not improve the agreement with experiment for the 28 S2NH order parameters, from 12 deviations larger than 0.1 in the *MD*_*2VB1* simulation to 11 deviations in the *MD*_*2VB1*_*Nres* simulation. Combining S2CH and S2NH order‐parameter restraining yields almost equally good agreement.

**Table 10:** Number of deviations, |*S* ^2^(exp)−*S* ^2^(MD)|, for the 11 S2NH values of Trp and Arg residues in the seven MD simulations.

Simulation	Size of *S* ^2^ deviation					
	0.05–0.1	0.1–0.2	0.2–0.3	0.3–0.4	0.4–0.5	>0.5
MD_2VB1	2	3	0	1	0	0
MD_4LZT	5	3	1	0	0	0
MD_1IEE	3	1	2	1	0	0
MD_1AKI	4	4	1	0	0	0
MD_2VB1_Cres	5	3	1	1	0	0
MD_2VB1_Nres	5	1	0	0	0	0
MD_2VB1_C+Nres	4	1	0	0	0	0

**Table 11:** Number of deviations, |*S* ^2^(exp)−*S* ^2^(MD)|, for the 17 S2NH2 values of Asnand Gln residues in the seven MD simulations.

Simulation	Size of *S* ^2^ deviation					
	0.05–0.1	0.1–0.2	0.2–0.3	0.3–0.4	0.4–0.5	>0.5
MD_2VB1	6	5	2	1	0	0
MD_4LZT	5	6	2	0	0	0
MD_1IEE	6	5	1	1	0	0
MD_1AKI	5	6	1	1	0	0
MD_2VB1_Cres	5	4	2	0	0	0
MD_2VB1_Nres	8	1	0	0	0	0
MD_2VB1_C+Nres	8	2	0	0	0	0

The data in Tables 5 and 7 and 9–11 indicate some unreliability of S2CH order‐parameter restraining, which may have different experimental or computational sources: 1) Some S2CH (exp) values (Table 5) are very different for two vectors in the same side chain, for example, for Ile58 CG2 and CD (1.0 and 0.16) and less so for Leu25 CD1 and CD2 (1.0 and 0.609) and Ile55 CG2 and CD (0.739 and 0.323). This suggests an unlikely large difference in mobility for nearby C−H vectors. 2) Some residues in the protein interior show unexpectedly low S2CH (exp) values, indicating high mobility, for example Val99 (CG1 0.487 and CG2 0.517) with a solvent accessible area in the 2VB1 X‐ray structure of only 7 %. 3) Some residues at the surface of the protein show unexpectedly high S2NH (exp) values, indicating low mobility, for example Thr89 CG2 with S2CH (exp)=1.0 and a solvent accessible area in the 2VB1 X‐ray structure of 76 %. 4) As discussed in section 2.3, the S2CH order‐parameter restraining algorithm restrains the C−C vector adjacent to the three C−H vectors of a methyl group, of which the relaxation is measured experimentally. This procedure is based on the assumption that the rotational motion of the C−H vectors around the axis of symmetry of the CH_3_ group is uniform and decoupled from the motion of the symmetry axis itself (the C−CH_3_ vector). These assumptions need not be true. This suggests that S2CH order‐parameter restraining is less reliable than S2NH order‐parameter restraining, for which the latter assumptions are not invoked.

Table 12 shows the number of NOE distance bound violations in the four X‐ray crystal structures and the seven MD simulations for the 1630 NOE distance bounds specified in Table S1. S2CH order‐parameter restraining decreases the number of NOE bound violations larger than 0.1 nm from 42 in the unrestrained *2VB1* simulation to 34 in the *MD*_*2VB1*_*Cres* simulation. S2NH order‐parameter restraining reduces the number of NOE bound violations larger than 0.1 nm from 42 in the unrestrained *MD*_*2VB1* simulation to 36 in the *MD*_*2VB1*_*Nres* simulation, halving the number of violations larger than 0.2 nm from 13 to 7. Combining S2CH and S2NH order‐parameter restraining yields better agreement, with 30 violations larger than 0.1 nm, as well as worse agreement, with nine violations larger than 0.2 nm. *S* ^2^ order‐parameter restraining in MD simulation improves agreement with experimentally derived NOE atom‐atom distance bounds.

**Table 12:** Number of NOE distance bound violations in the four X‐ray crystal structures and the seven MD simulations. Number of NOE distance bounds: 1630.

Structure or	Size of NOE distance bound violation [nm]					
simulation	0.05–0.1	0.1–0.15	0.15–0.2	0.2–0.25	0.25–0.3	>0.3
2VB1	21	7	5	0	0	0
4LZT	20	7	4	0	0	0
1IEE	20	7	5	0	0	0
1AKI	15	10	4	0	0	0
MD_2VB1	44	18	11	5	3	5
MD_4LZT	41	13	13	5	3	5
MD_1IEE	43	20	13	8	3	5
MD_1AKI	44	15	14	2	3	8
MD_2VB1_Cres	40	18	8	4	2	2
MD_2VB1_Nres	36	19	10	4	2	1
MD_2VB1_C+Nres	42	14	7	4	4	1

Table 13 shows 26 side‐chain ^3^ *J*_HαHβ_ coupling values for side chains, for which *S* ^2^ order‐parameter values derived from experiment are available, as derived from NMR measurements^[23]^ as well as from the unrestrained and *S* ^2^ order‐parameter restraining MD simulations starting from the 2VB1 X‐ray crystal structure. In the unrestrained simulation, six ^3^ *J*_HαHβ_ coupling values differ more than 2 Hz from the experimentally derived values, four for side chains for which experimentally derived S2CH order‐parameter values are available and two for side chains for which experimentally derived S2NH order‐parameter values are available. S2CH order‐parameter restraining induces two more deviations of ^3^ *J*_HαHβ_‐couplings in side chains with S2CH order‐parameter values, while S2NH order‐parameter restraining induces four more deviations of ^3^ *J*_HαHβ_‐couplings in side chains with S2NH order‐parameter values. Combined S2CH and S2NH order‐parameter restraining also leads to four more deviations of ^3^ *J*_HαHβ_‐couplings, two in side chains with S2CH order‐parameter values and two in side chains with S2NH order‐parameter values. Overall, *S* ^2^ order‐parameter restraining in MD simulation did not improve the agreement with experiment for the 26 side‐chain ^3^ *J*_HαHβ_ coupling values. For some residues, Thr51 for example, the agreement improves, whereas for other residues, Val29 for example, the agreement worsens.

**Table 13:** Side‐chain ^3^ *J*_HαHβ_ coupling values (26, for side chains for which *S* ^2^ order‐parameter values derived from experiment are available), in Hz, derived from NMR measurements and from the unrestrained and order‐parameter restrained MD simulations starting from the 2VB1 X‐ray crystal structure. Experimental data is from Tables III and IV of ref. [23] and consists of values that could be stereo‐specifically assigned based on NMR data as well as of values that could not be stereospecifically assigned in this way (marked with *). For the latter, stereo‐specific assignment of the experimental values for the β_2_ and β_3_ hydrogens is based on the ^3^ *J*_HαHβ_ coupling values calculated from the four unrestrained MD simulations starting from the four X‐ray structures in case 4 or 3 of the unrestrained MD simulations suggested the same stereo‐specific assignment. The root‐mean‐square fluctuations (RMSF) of the ^3^ *J*_HαHβ_ couplings in the MD simulations are given within parentheses. MD values differing more than 2 Hz from the experimental value are denoted using italics.

Residue	Experimental	Unrestrained MD	Order‐parameter restrained MD		
	value	*MD*_*2VB1*	*2VB1*_*Cres*	*2VB1*_*Nres*	*2VB1*_*C+Nres*
Val2	10.8	9.3 (4.6)	*6.1* (4.6)	10.8 (3.8)	*7.4* (4.8)
Asn19 β_2_	7.3	8.3 (4.6)	8.4 (4.1)	8.9 (3.9)	7.5 (4.3)
β_3_	6.4	5.9 (4.6)	5.0 (4.5)	*4.3* (3.9)	5.1 (4.2)
Val29	11.1	10.1 (4.3)	*6.3* (4.6)	9.6 (4.4)	*3.0* (1.6)
Asn37* β_2_	8.1	9.1 (4.6)	6.6 (4.8)	*5.2* (4.3)	7.6 (4.8)
β_3_	4.2	5.1 (3.9)	*7.3* (4.6)	*8.8* (4.4)	*6.7* (4.5)
Thr43	3.7	3.4 (2.6)	4.6 (3.9)	3.1 (2.0)	5.1 (4.2)
Thr47	2.6	3.0 (1.5)	4.0 (3.2)	2.9 (1.4)	3.9 (3.1)
Thr51	9.3	*5.6* (4.6)	9.1 (4.6)	8.8 (4.7)	9.4 (4.2)
Asn65* β_2_	4.5	4.2 (3.1)	3.2 (1.2)	3.3 (2.2)	3.4 (2.4)
β_3_	11.4	11.3 (3.2)	12.4 (0.9)	10.5 (3.4)	10.4 (3.4)
Thr69	9.3	*6.1* (4.6)	*12.6* (0.5)	*12.4* (0.7)	*12.6* (0.5)
Asn74* β_2_	10.5	11.3 (3.2)	11.9 (2.3)	*3.1* (1.4)	*2.9* (1.5)
β_3_	3.9	4.0 (2.2)	3.9 (1.9)	*6.0* (3.7)	*7.6* (4.2)
Leu75 β_2_	12.4	11.5 (2.4)	11.7 (2.1)	10.4 (3.3)	10.9 (2.9)
β_3_	2.1	3.0 (1.8)	2.9 (1.8)	3.3 (2.5)	3.1 (2.1)
Asn77* β_2_	8.3	*10.8* (3.4)	*11.0* (3.4)	*10.6* (3.7)	*10.6* (3.8)
β_3_	5.9	*3.8* (2.6)	4.3 (3.2)	4.4 (3.4)	4.4 (3.4)
Ile88	4.5	4.3 (3.8)	3.2 (2.3)	*2.4* (0.9)	4.2 (3.6)
Thr89	9.5	*4.8* (3.4)	*6.9* (4.9)	*3.5* (2.7)	*3.0* (1.0)
Val92	10.1	9.6 (4.5)	*4.0* (2.7)	*12.3* (1.5)	11.8 (2.4)
Asn93* β_2_	10.8	10.7 (3.6)	9.9 (4.1)	10.6 (3.6)	9.4 (4.4)
β_3_	3.5	4.1 (3.6)	4.8 (4.2)	4.4 (3.7)	5.4 (4.3)
Val99	6.3	*3.0* (1.6)	5.5 (4.3)	6.4 (4.6)	*3.7* (3.3)
Val109	8.0	9.0 (4.7)	*5.1* (4.3)	6.4 (4.8)	*5.9* (4.6)
Ile124	4.6	4.1 (2.7)	3.5 (1.4)	5.2 (3.8)	3.3 (1.6)

Overall, the structure of HEWL is maintained in all seven MD simulations, as is indicated in Figures S1 and S2 showing the backbone atom‐positional root‐mean‐square deviation (RMSD) from the 2VB1 X‐ray structure as function of time.

## 3 Conclusions

*S* ^2^ order parameters for C−H and N−H bonds in proteins derived from NMR relaxation measurements reflect the directional mobility of these bonds. Consequently, they cannot be related to a single protein structure, but to an ensemble of such structures. Therefore, *S* ^2^ order parameters are not used as data for standard single‐structure determination of proteins. A comparison of four X‐ray structures of HEWL and four MD simulations starting from these four different X‐ray structures illustrates the need of a conformational ensemble representation of the HEWL protein, in particular for its side chains. MD simulation allows for averaging over an ensemble of trajectory structures, which is used in protein structure determination based on *S* ^2^ order parameters.^[9]^ S2NH order‐parameter restraining can be directly applied to the N−H bond vectors in a protein. In contrast, S2CH order‐parameter restraining of C−H bond vectors in methyl groups makes no sense because of the fast rotation of the three C−H bonds around their symmetry axis parallel to the C−CH_3_ bond. By assuming the above‐mentioned rotation to be uniform and independent from the motion of the symmetry axis itself,^[15]^ the S2CH order‐parameter restraining algorithm can be applied to the C−CH_3_ bond. The results for HEWL show that S2CH order‐parameter restraining is more problematic than S2NH order‐parameter restraining, which may be due to less valid assumptions and approximations used to derive experimental S2CH (exp) values from NMR relaxation measurements and the assumptions of uniform rotational motion of methyl C−H bonds around their symmetry axis and of the independence of these motions from each other.

The application of *S* ^2^ order‐parameter restraining to the protein HEWL shows that this technique is able to produce a conformational ensemble compatible with the experimentally derived S2CH (exp) and S2NH (exp) values. *S* ^2^ order‐parameter restraining in MD simulation does improve the agreement with 1630 NOE atom‐atom distance bounds for HEWL. It maintains the overall structure of the protein and induces slightly more mobility, reflected in the backbone atom‐positional fluctuations. The unrestrained MD simulations show a high level of conformational disorder for side chains on the protein surface. However, this disorder is increased even further on *S* ^2^ order‐parameter restraining. In the MD simulations, which show good agreement with the experimental order parameters, the populations of many of the hydrogen bonds that are seen in all or most of the X‐ray structures are low. This has important implications for the use of X‐ray structure data in areas such as drug design, the interpretation of mutational data and receptor binding studies.

## Conflict of interest

The authors declare no conflict of interest.

## Supporting information

As a service to our authors and readers, this journal provides supporting information supplied by the authors. Such materials are peer reviewed and may be re‐organized for online delivery, but are not copy‐edited or typeset. Technical support issues arising from supporting information (other than missing files) should be addressed to the authors.

Supplementary

## References

[1] W. F. van Gunsteren , J. R. Allison , X. Daura , J. Dolenc , N. Hansen , A. E. Mark , C. Oostenbrink , V. H. Rusu , L. J. Smith , Angew. Chem. Int. Ed. 2016, 55, 15990–16010; 10.1002/anie.201601828 27862777

[2] J. Fennen , A. E. Torda , W. F. van Gunsteren , J. Biomol. NMR 1995, 6, 163–170. 8589605 10.1007/BF00211780

[3] A. E. Torda , R. M. Scheek , W. F. van Gunsteren , Chem. Phys. Lett. 1989, 157, 289–294.

[4] P. Gros , W. F. van Gunsteren , W. G. J. Hol , Science 1990, 249, 1149–1152. 2396108 10.1126/science.2396108

[5] C. A. Schiffer , W. F. van Gunsteren , Proteins Struct. Funct. Genet. 1999, 36, 501–511. 10450092

[6] A. E. Torda , R. M. Scheek , W. F. van Gunsteren , J. Mol. Biol. 1990, 214, 223–235. 2370663 10.1016/0022-2836(90)90157-H

[7] C. A. Schiffer , R. Huber , K. Wüthrich , W. F. van Gunsteren , J. Mol. Biol. 1994, 241, 588–599. 7520085 10.1006/jmbi.1994.1533

[8] A. E. Torda , R. M. Brunne , T. Huber , H. Kessler , W. F. van Gunsteren , J. Biomol. NMR 1993, 3, 55–66. 8448435 10.1007/BF00242475

[9] N. Hansen , F. Heller , N. Schmid , W. F. van Gunsteren , J. Biomol. NMR 2014, 60, 169–187. 25312596 10.1007/s10858-014-9866-7

[10] G. Lipari , A. Szabo , J. Am. Chem. Soc. 1982, 104, 4546–4559.

[11] E. R. Henry , A. Szabo , J. Chem. Phys. 1985, 82, 4753–4761.

[12] E. T. Olejniczak , C. M. Dobson , M. Karplus , R. M. Levy , J. Am. Chem. Soc. 1984, 106, 1923–1930.

[13] I. Chandrasekhar , G. M. Clore , A. Szabo , A. M. Gronenborn , B. R. Brooks , J. Mol. Biol. 1992, 226, 239–250. 1619653 10.1016/0022-2836(92)90136-8

[14] L. J. Smith , A. E. Mark , C. M. Dobson , W. F. van Gunsteren , Biochemistry 1995, 34, 10918–10931. 7662673 10.1021/bi00034a026

[15] D. C. Chatfield , A. Szabo , B. R. Brooks , J. Am. Chem. Soc. 1998, 120, 5301–5311.

[16] V. Kasinath , K. A. Sharp , A. J. Wand , J. Am. Chem. Soc. 2013, 135, 15092–15100. 24007504 10.1021/ja405200u PMC3821934

[17] E. S. O'Brien , A. J. Wand , K. A. Sharp , Prot. Sci. 2016, 25, 1156–1160. 10.1002/pro.2922 PMC4941777 26990788

[18] L. J. Smith , W. F. van Gunsteren , N. Hansen , J. Phys. Chem. B 2017, 121, 7055–7063. 28640620 10.1021/acs.jpcb.7b03647

[19] L. J. Smith , R. Athill , W. F. van Gunsteren , N. Hansen , Chem. Eur. J. 2017, 23, 9585–9591. 28503764 10.1002/chem.201700896

[20] M. Buck , J. Boyd , C. Redfield , D. A. MacKenzie , D. J. Jeenes , D. B. Archer , C. M. Dobson , Biochemistry 1995, 34, 4041–4055. 7696270 10.1021/bi00012a023

[21] V. Moorman , K. G. Valentine , A. J. Wand , Prot. Sci. 2012, 21, 1066–1073. 10.1002/pro.2092 PMC3403443 22593013

[22] L. J. Smith , M. J. Sutcliffe , C. Redfield , C. M. Dobson , J. Mol. Biol. 1993, 229, 930–944. 8445657 10.1006/jmbi.1993.1097

[23] L. J. Smith , M. J. Sutcliffe , C. Redfield , C. M. Dobson , Biochemistry 1991, 30, 986–996. 1989688 10.1021/bi00218a015

[24] V. A. Higman , J. Boyd , L. J. Smith , C. Redfield , J. Biomol. NMR 2004, 30, 327–346. 15754058 10.1007/s10858-004-3218-y

[25] D. Steiner , J. R. Allison , A. P. Eichenberger , W. F. van Gunsteren , J. Biomol. NMR 2012, 53, 223–246. 22714630 10.1007/s10858-012-9634-5

[26] V. A. Higman , J. Boyd , L. J. Smith , C. Redfield , J. Biomol. NMR 2011, 49, 53–60. 21184138 10.1007/s10858-010-9457-1 PMC3020303

[27] N. Schmid , C. D. Christ , M. Christen , A. P. Eichenberger , W. F. van Gunsteren , Comp. Phys. Comm. 2012, 183, 890–903.

[28] N. Schmid , J. R. Allison , J. Dolenc , A. P. Eichenberger , A.-P. E. Kunz , W. F. van Gunsteren , J. Biomol. NMR 2011, 51, 265–281. 21858640 10.1007/s10858-011-9534-0

[29] W. F. van Gunsteren, et al., http://www.gromos.net, GROMOS.

[30] D. Poger , W. F. van Gunsteren , A. E. Mark , J. Comput. Chem. 2010, 31, 1117–1125. 19827145 10.1002/jcc.21396

[31] N. Schmid , A. P. Eichenberger , A. Choutko , S. Riniker , M. Winger , A. E. Mark , W. F. van Gunsteren , Eur. Biophys. J. 2011, 40, 843–856. 21533652 10.1007/s00249-011-0700-9

[32] K. Bartik , C. Redfield , C. M. Dobson , Biophys. J. 1994, 66, 1180–1184. 8038389 10.1016/S0006-3495(94)80900-2 PMC1275825

[33] H. J. C. Berendsen , J. P. M. Postma , W. F. van Gunsteren , J. Hermans in Intermolecular Forces (Ed.: B. Pullman ), Reidel, Dordrecht, 1981, pp. 331–342.

[34] J. P. Ryckaert , G. Ciccotti , H. J. C. Berendsen , J. Comput. Phys. 1977, 23, 327–341.

[35] R. W. Hockney , J. W. Eastwood , Computer Simulation Using Particles, McGraw-Hill, New York, 1981.

[36] W. F. van Gunsteren , H. J. C. Berendsen , R. G. Geurtsen , H. R. J. Zwinderman , Ann. New York Acad. Sci. 1986, 482, 287–303. 3471112 10.1111/j.1749-6632.1986.tb20962.x

[37] J. A. Barker , R. O. Watts , Mol. Phys. 1973, 26, 789–792.

[38] I. G. Tironi , R. Sperb , P. E. Smith , W. F. van Gunsteren , J. Chem. Phys. 1995, 102, 5451–5459.

[39] T. N. Heinz , W. F. van Gunsteren , P. H. Hünenberger , J. Chem. Phys. 2001, 115, 1125–1136.

[40] H. M. Berman , J. Westbrook , Z. Feng , G. Gilliland , T. N. Bhat , H. Weissig , I. N. Shindyalov , P. E. Bourne , Nucleic Acids Res. 2000, 28, 235–242. 10592235 10.1093/nar/28.1.235 PMC102472

[41] H. J. C. Berendsen , J. P. M. Postma , W. F. van Gunsteren , A. DiNola , J. R. Haak , J. Chem. Phys. 1984, 81, 3684–3690.

[42] A. P. Eichenberger , J. R. Allison , J. Dolenc , D. P. Geerke , B. A. C. Horta , K. Meier , C. Oostenbrink , N. Schmid , D. Steiner , D. Wang , W. F. van Gunsteren , J. Chem. Theory Comput. 2011, 7, 3379–3390. 26598168 10.1021/ct2003622

[43] W. F. van Gunsteren , R. Boelens , R. Kaptein , R. M. Scheek , E. R. P. Zuiderweg in Molecular Dynamics and Protein Structure (Ed.: J. Hermans ), Polycrystal Book Service, Western Springs, 1985, pp 92–99.

[44] K. Wüthrich , M. Billeter , W. Braun , J. Mol. Biol. 1983, 169, 949–961. 6313936 10.1016/s0022-2836(83)80144-2

[45] A. E. Torda , W. F. van Gunsteren , Comp. Phys. Commun. 1991, 62, 289–296.

[46] M. Karplus , J. Chem. Phys. 1959, 30, 11–15.

[47] M. Karplus , J. Amer. Chem. Soc. 1963, 85, 2870–2871.

[48] A. deMarco , M. Llinás , K. Wüthrich , Biopolymers 1978, 17, 617–636.

[49] W. Kabsch , C. Sander , Biopolymers 1983, 22, 2577–2637. 6667333 10.1002/bip.360221211

